# An Amide Alkaloid Isolated from *Ephedra sinica* Ameliorates OVA-Induced Allergic Asthma by Inhibiting Mast Cell Activation and Dendritic Cell Maturation

**DOI:** 10.3390/ijms232113541

**Published:** 2022-11-04

**Authors:** Jufang Jia, Mengnan Zeng, Denghui Zhu, Xinmian Jiao, Beibei Zhang, Ruolan Yang, Weisheng Feng, Xiaoke Zheng

**Affiliations:** 1 College of Pharmacy, Henan University of Chinese Medicine, Zhengzhou 450046, China; 2 The Engineering and Technology Center for Chinese Medicine Development of Henan Province, Zhengzhou 450046, China; 3 Collaborative Innovation Center for Respiratory Disease Diagnosis and Treatment & Chinese Medicine Development of Henan Province, Zhengzhou 450046, China

**Keywords:** allergic asthma, alkaloid, immunosuppression, mast cells, dendritic cells

## Abstract

Asthma, which is a chronic inflammatory disease of the airways, is usually caused by allergens in which various structures and immune cells are involved. *Ephedra sinica*, the most commonly used Chinese medicine, has significant clinical effects on asthma, but its components are complex and the mechanism of action has not been fully elucidated. Among its components, we identified an amide alkaloid (EB-A) and investigated its anti-asthmatic activity and the underlying mechanisms. In this study, we replicated an OVA-sensitized/challenged allergic asthma mouse model, and divided the mice into a model (OVA) group, positive drug (Y, 0.5 mg/kg/day) group, and EB-A treatment with low (Low, 10 mg/kg/day) and high dose (High, 20 mg/kg/day) groups. Asthma-related features were analyzed through the airway hyperresponsiveness (AHR), cough and wheeze indexes, allergen-specific IgE, prostaglandin D2 (PDG2), and lung histology in mice. The levels of apoptosis and reactive oxygen species (ROS) in the primary lung cells, cytokines in the serum and broncho-alveolar lavage fluid (BALF), and proteinase-activated receptor-2 (PAR2) pathway activation in the lung tissue were measured to evaluate the inflammatory injury and lung epithelial barrier damage in the mice. Dendritic cell (DC) maturation and mast cell (MC) activation were verified in vitro and in vivo. Furthermore, the effect of a PAR2 activation in lung epithelial cells on the maturation of DCs was evaluated by the co-culture system of (human bronchial epithelial cell lines) 16HBE and bone marrow-derived dendritic cells (BMDCs). The results showed that EB-A inhibited the typical asthmatic phenotypes, as well as lung injury and inflammation, MC activation and degranulation, and DC maturation in the OVA-sensitized/challenged BALB/c mice. In addition, EB-A inhibited the expression of PAR2 in the lung epithelial cells and significantly interfered with the maturation of DCs after inhibiting PAR2. Taken together, our study firstly demonstrated that EB-A could ameliorate OVA-induced allergic asthma by inhibiting MC activation and DC maturation, and the molecular mechanism of EB-A’s anti-asthmatic activity might be mediated by inhibiting PAR2. Our data provide a molecular justification for the use of EB-A in the treatment of allergic asthma.

## 1. Introduction

Allergic asthma is a common chronic heterogeneous disease of the lower airways, and is characterized by a reversible airflow obstruction with airway inflammation and hyperresponsiveness, and symptoms such as cough, wheezing, dyspnea, and chest tightness during an asthma attack. It affects more than 300 million people globally, according to the data from the Global Initiative for Asthma (GINA), with a rapid growth rate of 20–25% per decade, and the number of people suffering from asthma worldwide is expected to increase to 400 million by 2025 [1]. In China, asthma causes substantial health and economic burdens, affecting about 45.7 million people older than 20 years; among them, nearly 70% are not diagnosed and 95% do not receive standard treatment [2]. Inhaled corticosteroids (ICS) have been the basis for asthma treatment; however, many patients with asthma are resistant to inhaled or systemic corticosteroids, leaving few treatment options for them [3]. Therefore, there is an urgent need to develop new therapies and drugs to control the symptoms and exacerbations in severe asthma patients and to avoid adverse reactions caused by the administration of oral corticosteroids (OCS).

The pathophysiology of asthma involves changes in a variety of immune and structural cells. Epithelial barrier injury has been shown to play an important role in the occurrence and development of many allergic and autoimmune diseases such as asthma, allergic rhinitis, atopic dermatitis, and inflammatory bowel disease [4]. Airway epithelial cells express pattern-recognition receptors, such as Toll-like receptors (TLRs) and proteinase-activated receptors (PARs), which can sense a wide range of environmental stimuli, including allergens, microorganisms, and particulate contaminants [5,6]. When stimulated by inhalation, the epithelial cells release proinflammatory cytokines and activate DCs, which in turn promote Th2 development and drive the activation of innate immune cells [7,8]. As the most powerful antigen-presenting cells (APCs), DCs play important roles in initiating and directing immune responses. After the immune response is initiated, a variety of immune cells such as group 2 innate lymphoid cells (ILC2), B cells, MCs, and eosinophils are involved in the reaction process. In particular, MCs are key effector cells in the pathogenesis of asthma, and their released granules can cause smooth muscle contraction and mucosal edema, thereby leading to bronchiolar stenosis [9,10]. Currently, many therapeutic drugs target this critical process of degranulation.

Traditional Chinese medicine recipes, such as Ma-Xing-Shi-Gan Decoction, Xiaoqinglong Decoction, and Shegan–Mahuang Decoction, are widely used in the treatment of asthma; *Ephedra sinica* is the main component of these formulations. Modern pharmacological studies show that *Ephedra sinica* has excellent medicinal value, with the effects of sweating to dissipate cold, lung dispersing to relieve asthma, inducing diuresis to alleviate edema, lowering blood sugar, as well as anti-inflammatory and immunomodulatory effects [11,12]. As a clinical “special medicine for lung”, *Ephedra sinica* is often used in the treatment of lung diseases. In a previous study on the chemical composition of *Ephedra equisetina* Bge. (a species of *Ephedra sinica*), we found an amide alkaloid (EB-A), which could inhibit the degranulation of RBL-2H3 induced by compound 48/80, suggesting that EB-A may have anti-asthmatic activity (unpublished data). In the current experiment, we focused on investigating the ameliorative effect of EB-A on asthmatic mice and the mechanism of its immunomodulatory effect, so as to provide experimental evidence for the pharmacological study of *Ephedra sinica* and the development of anti-asthma drugs.

## 2. Results

### 2.1. Structure and PLC Analysis of EB-A

**Figure 1:**
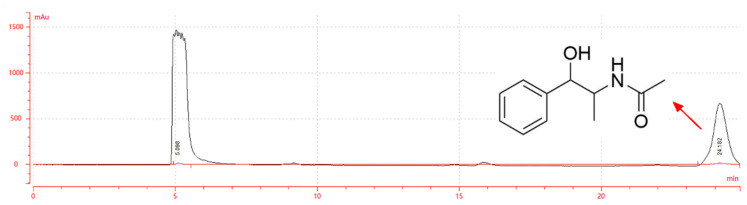
Liquid chromatogram and structural formula of EB-A.

The structural formula and liquid chromatogram of EB-A are shown in Figure 1. When the retention time was 24.182 min, a single peak appeared, which represented EB-A [13].

### 2.2. EB-A Ameliorates OVA-Sensitized/Challenged BALB/c Mice Exhibiting a Typical Asthmatic Phenotype

**Figure 2:**
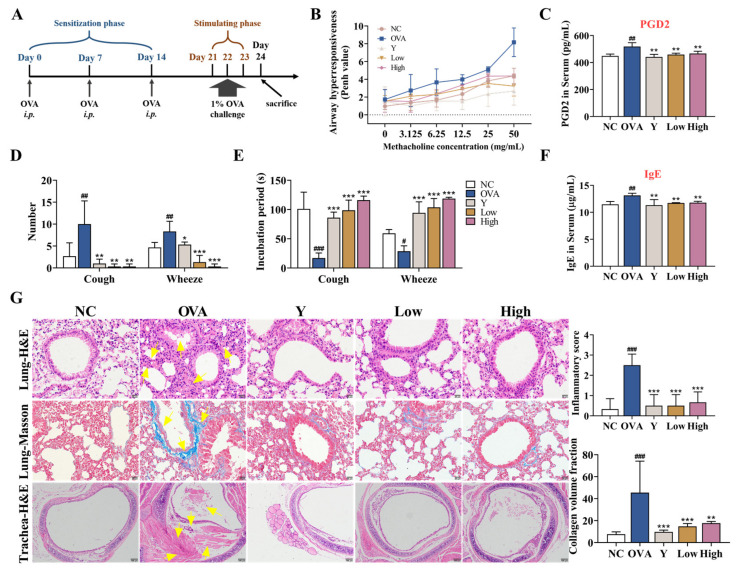
EB-A ameliorates asthma features on a mouse model of asthma. (**A**) Schematic representation of a mouse model of OVA-induced allergic asthma. The “*i.p.*” stands for intraperitoneal injection. (**B**) Airway hyperresponsiveness was measured as methacholine-induced increases in transpulmonary resistance in mice. Data are expressed as a mean value of percentage increased from the baseline of the transpulmonary resistance ± SD values of three mice (white or flesh pink: NC, peacock blue: OVA, powder blue: Y, lawn green: Low, chartreuse: High). (**C**) Serum PGD2 levels were measured using ELISA. (**D**,**E**) Twenty-four hours after the last administration, the number and incubation period of the cough and wheeze indexes were detected in the mice. (**F**) Serum IgE levels were measured using ELISA. (**G**) HE and Masson staining was performed, and then the inflammation intensity and collagen fiber area were scored. The yellow arrow indicates the lesion (400 × or 100 ×). All data are shown as mean ± SD. ^#^ *p* < 0.05, ^##^ *p* < 0.01, ^###^ *p* < 0.001, compared with the NC group; * *p* < 0.05, ** *p* < 0.01, *** *p* < 0.001, compared with the OVA group.

To investigate the therapeutic potential of EB-A on allergic asthma, we replicated an OVA-sensitized/challenged allergic asthma mouse model (Figure 2A). Initially, the AHR was measured in the mice after inoculation with methacholine. As expected, the asthmatic mice displayed an increase in lung resistance (expressed in Penh) in response to methacholine compared with that of the control mice (*n* = 3, Figure 2B). Correspondingly, the content of prostaglandin D2 (PGD2), a cytokine that can cause bronchial smooth muscle contraction, increased in the serum of the asthmatic mice (*p* < 0.01) (Figure 2C). In contrast, in the mice receiving EB-A, we observed a decrease in the Penh and PGD2 compared with the asthmatic mice to a control level (*p* < 0.01) (Figure 2B,C). In the cough and wheeze test, the numbers of cough and asthma in the asthmatic mice were significantly increased (*p* < 0.01), and the incubation period of the cough and wheeze indexes was appreciably shortened (*p* < 0.001, or *p* < 0.05). EB-A significantly reversed this change (*p* < 0.001, *p* < 0.01, or *p* < 0.05) (Figure 2D,E). In addition, the level of total IgE in the serum of the OVA group was significantly higher than that of the NC group (*p* < 0.01), while those of the Y group and the EB-A-Low and High group were significantly lower than that of the OVA group (*p* < 0.01) (Figure 2F). Then, pulmonary lesions were investigated by histology. The histological analysis revealed that the lungs of the asthmatic mice were severely inflamed, with extensive inflammatory cell infiltration and a significantly increased collagen deposition (*p* < 0.001). The EB-A remarkably reduced the pulmonary lesions (*p* < 0.001) based on the lung histological scoring (Figure 2G). Overall, our results demonstrated that EB-A considerably reduced the global asthma characteristics, reduced airway hyperresponsiveness, prevented changes in the number and incubation period of cough and wheeze indexes, and attenuated the inflammatory infiltration and collagen fibers deposition; therefore, EB-A displays a high possibility for success in the treatment of allergic asthma.

### 2.3. EB-A Alleviates Lung Injury and Inflammation in OVA-Induced Mice

**Figure 3:**
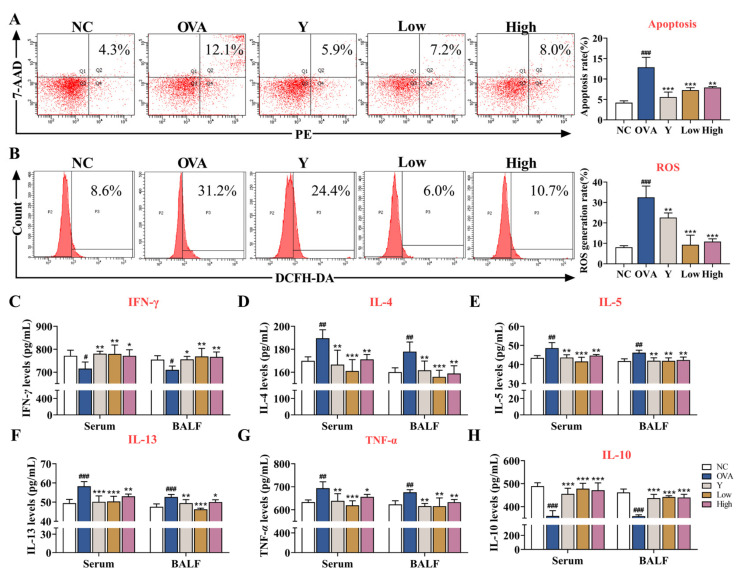
EB-A alleviated lung injury and inflammation in OVA-induced mice. (**A**) The levels of apoptosis in lung cells were tested by flow cytometry. (**B**) The levels of ROS in lung cells were tested by flow cytometry. (**C**–**H**) Graphs show levels of IFN-γ, IL-4, IL-5, IL-13, TNF-*α*, and IL-10 in serum and BALF. All data are shown as mean ± SD. ^#^ *p* < 0.05, ^##^ *p* < 0.01, ^###^ *p* < 0.001, compared with the NC group; * *p* < 0.05, ** *p* < 0.01, *** *p* < 0.001, compared with the OVA group.

To further evaluate the effect of EB-A on lung tissue damage and inflammation in asthmatic mice, we detected the apoptosis and ROS levels in primary mouse lung tissue cells, as well as the levels of various cytokines in the serum and BALF. First, we examined the apoptosis and ROS levels in primary mouse lung tissue cells using FCM. The results showed that the levels of apoptosis and ROS in the lung tissue of the asthmatic mice significantly increased (*p* < 0.001), while EB-A significantly decreased the levels of apoptosis and ROS (*p* < 0.01) (Figure 3A,B). Furthermore, the cytokine levels of serum and BALF were detected by ELISA. Levels of the analyzed cytokines (including IL-4, IL-5, IL-13, and TNF-*α*), except for those of IFN-γ and IL-10, which were downregulated, were upregulated after an allergic provocation in the mice (*p* < 0.001, *p* < 0.01, or *p* < 0.05). In sharp contrast, the levels of IL-4, IL-5, IL-13, and TNF-*α* of the Y group and the EB-A-Low and High group significantly decreased, compared with those in the OVA group, while changes in IFN-γ and IL-10 showed the opposite trend (*p* < 0.001, *p* < 0.01, or *p* < 0.05) (Figure 3C–H). As noted above, EB-A, surprisingly, alleviated lung injury and inflammation in the OVA-induced mice.

### 2.4. EB-A Treatment Inhibits the Activation of PAR2 in Lung Tissue In Vivo

**Figure 4:**
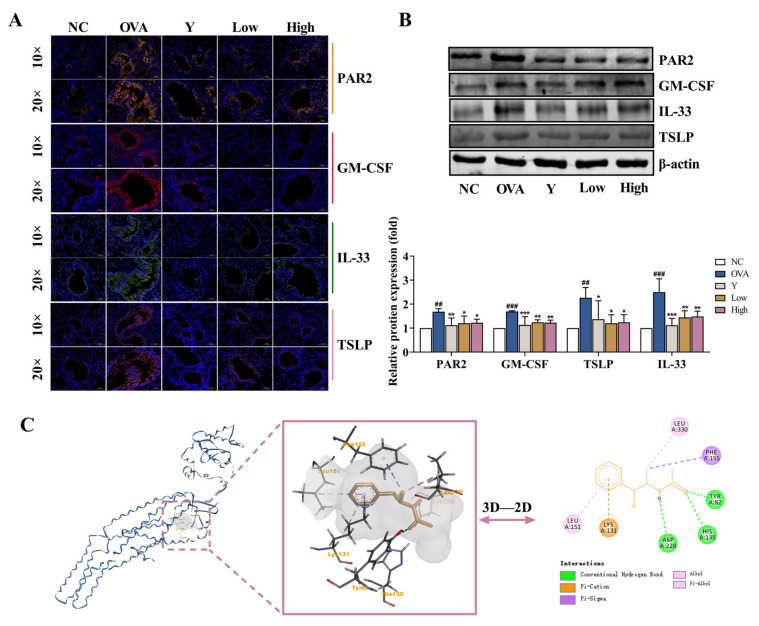
EB-A treatment inhibited the activation of protease-activated receptor-2 (PAR2) in lung tissue in vivo. (**A**) The expressional levels of PAR2, GM-CSF, IL-33, and TSLP were detected by immunofluorescence staining. (**B**) The expressional levels of PAR2, GM-CSF, IL-33, and TSLP were detected by Western blotting. (**C**) The binding conformation of EB-A in the PAR2 antagonist pocket was predicted by molecular docking. All data are shown as mean ± SD. ^##^ *p* < 0.01, ^###^ *p* < 0.001, compared with the NC group; * *p* < 0.05, ** *p* < 0.01, *** *p* < 0.001, compared with the OVA group.

PAR2 is a G-coupled receptor involved in inflammatory reactions [14]. In this study, we observed that PAR2 was activated in the lung tissue of asthmatic mice, and the expressional levels of the downstream-related effector molecules, including GM-CSF, IL-33, and TSLP, significantly increased (*p* < 0.001, or *p* < 0.01); however, the activation of PAR2 was remarkably inhibited in the lung tissue of the EB-A-treated mice. The levels of GM-CSF, IL-33, and TSLP also significantly decreased (*p* < 0.001, *p* < 0.01, or *p* < 0.05) (Figure 4A,B). This suggests that an EB-A treatment inhibits the activation of PAR2 in lung tissue. The original Western blots images and data are shown in Figure S1 and Table S1. To further investigate the intermolecular interaction between EB-A and PAR2, molecular docking was performed between PAR2 (PDB ID: 5NDD) and EB-A. As shown in Figure 4C, hydrogen bonds and electrostatic and hydrophobic interactions occurred between the EB-A and the antagonist binding pocket in the PAR2 [15], suggesting that EB-A is a potential PAR2 antagonist.

### 2.5. EB-A Treatment Inhibits Dendritic Cell Maturation after OVA Sensitization In Vivo

**Figure 5:**
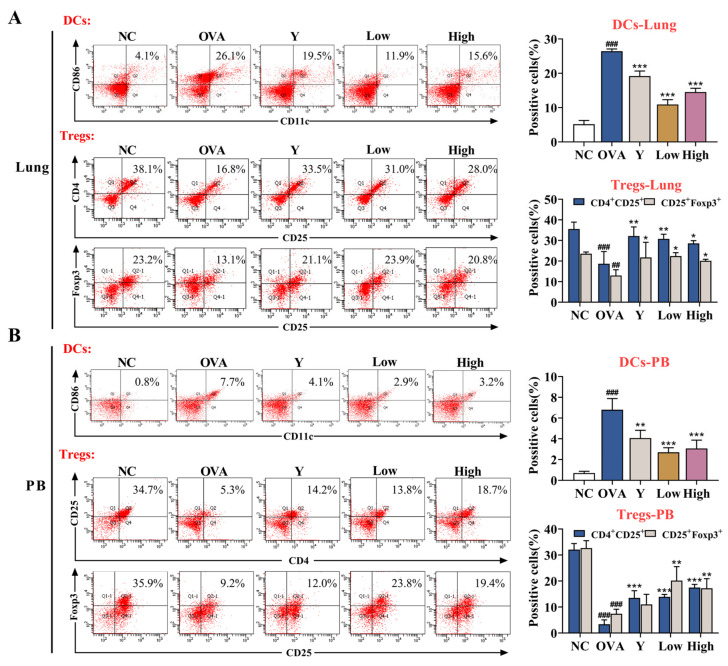
EB-A treatment decreases dendritic cells maturation and increases regulatory T cells differentiation after OVA sensitization in vivo. (**A**) DCs and Tregs in mouse lung tissues were detected by FCM. (**B**) DCs and Tregs in the peripheral blood of mice were detected by FCM. All data are shown as mean ± SD. ^##^ *p* < 0.01, ^###^ *p* < 0.001, compared with the NC group; * *p* < 0.05, ** *p* < 0.01, *** *p* < 0.001, compared with the OVA group.

Although lung type 2 inflammatory disease can be triggered by a wide range of stimuli, DCs are centrally involved in the initiation and direction of the adaptive immune response and immunopathology that ultimately determines the severity of chronic disease [16]. In an inflammatory environment, DCs tend to mature, and then the expression of co-stimulatory molecules on the surface of mature DCs (mDCs) is upregulated, and their immunogenicity is enhanced [17]. To assess the role of EB-A in DC maturation, we detected the levels of DCs in mouse peripheral blood (PB) and lung tissue. The levels of Tregs were also examined for subsequent effects of inhibiting DC maturation on immune tolerance. As shown in Figure 5, the EB-A treatment decreased the DC maturation and increased the Treg differentiation after OVA sensitization in vivo (*p* < 0.001, *p* < 0.01, or *p* < 0.05).

### 2.6. EB-A Inhibits Mast Cell Activation and Degranulation In Vivo and In Vitro

**Figure 6:**
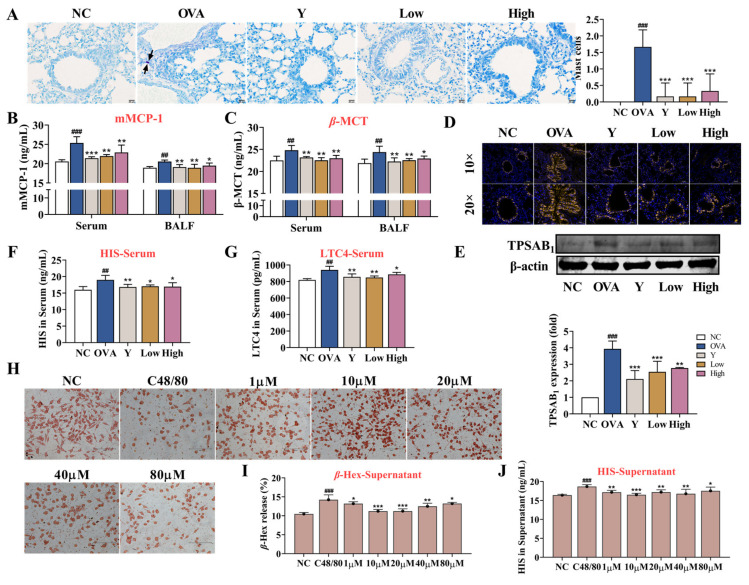
EB-A inhibits mast cell activation and degranulation in vivo and in vitro. (**A**) Mast cells were revealed by toluidine blue staining and counted (at least three random fields per tissue section). The black arrow indicates the mast cells. (**B**,**C**) mMCP-1 and *β*-MCT levels in serum and BALF were measured using ELISA. (**D**,**E**) TPSAB1 protein expression level was, respectively, measured by immunofluorescence staining and Western blotting. (**F,G**) Histamine and LTC4 levels in serum were measured using ELISA. (**H**) Neutral red staining solution was used to stain degranulation in RBL-2H3 cells. (**I**) The release of *β*-Hexosaminidase (*β*-Hex) from RBL-2H3 cells was detected by a substrate chromogenic assay. (**J**) The release of histamine from RBL-2H3 cells was detected by ELISA. All data are shown as mean ± SD. ^##^ *p* < 0.01, ^###^ *p* < 0.001, compared with the NC group; * *p* < 0.05, ** *p* < 0.01, *** *p* < 0.001, compared with the OVA group.

Decades of research have implicated mast cells and their mediators in the pathogenesis of asthma and allergy [18]. MCs are key players in the asthmatic response through the secretion of a multitude of mediators with proinflammatory and airway-constrictive effects, such as histamine and leukotriene C4. In the present study, we examined the activation of MCs in mouse lung tissue. Toluidine blue staining showed that MCs were hardly seen in normal mouse lung tissues, while MCs and scattered staining particles in the lung interstitial were observed in OVA-induced asthma mice lung tissues (*p* < 0.001) (Figure 6A). Moreover, the ELISA and lung tissue immunofluorescence data showed that the levels of mast cell proteinase 1 (mMCP-1) and *β*-trypsin (*β*-MCT) in the serum and BALF (Figure 6B,C), histamine and LTC4 in the serum (Figure 6F,G), and the expression of TPSAB1 in the lung tissue of asthmatic mice (Figure 6D,E) significantly increased, which suggested the activation and degranulation behavior of the MCs. In contrast, the EB-A treatment obviously inhibited the MCs activation (*p* < 0.001, *p* < 0.01, or *p* < 0.05). Similarly, the EB-A treatment inhibited the compound 48/80-induced degranulation of RBL-2H3 cells in vitro (*p* < 0.001, *p* < 0.01, or *p* < 0.05) (Figure 6H–J). This reflects that EB-A could inhibit mast cell activation and degranulation in vivo and in vitro.

### 2.7. EB-A Treatment on DCs In Vitro

**Figure 7:**
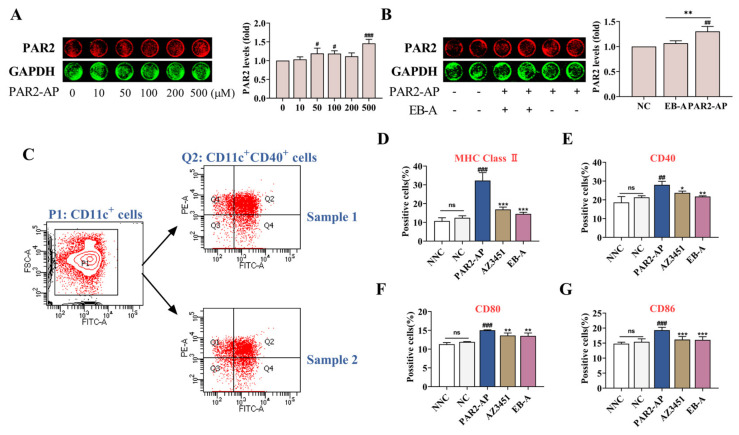
EB-A prevents DCs maturation by inhibiting PAR2 activation in vitro. (**A**) In-cell Western blot was used to evaluate the effects of different concentrations of PAR2-AP on the expression of PAR2 in 16HBE cells. (**B**) The effect of EB-A on PAR2 expression in 16HBE cells was estimated by in-cell Western blot. (**C**) Logic diagram of flow gate, taking CD40 as an example. (**D**) The levels of MHC Class Ⅱ on the surface of BMDCs in the co-culture system were detected by FCM. (**E**) The levels of CD40 on the surface of BMDCs in the co-culture system were detected by FCM. (**F**) The levels of CD80 on the surface of BMDCs in the co-culture system were detected by FCM. (**G**) The levels of CD86 on the surface of BMDCs in the co-culture system were detected by FCM. All data are shown as mean ± SD. ^#^ *p* < 0.05, ^##^ *p* < 0.01, ^###^ *p* < 0.001, compared with the NC group; * *p* < 0.05, ** *p* < 0.01, *** *p* < 0.001, compared with the PAR2-AP treated group.

In vivo results showed that PAR2 was activated in the lung tissue of the asthmatic mice and, consistent with the existing studies [4,19], the resulting epithelial cytokine response subsequently stimulated a DC activation. To explore the effect of PAR2 activation in lung epithelial cells on DC maturation, we established a co-culture system of 16HBE cells and BMDCs in vitro. As shown in Figure 7A,B, at the concentration of 500 mM, PAR2-activating peptide (PAR2-AP) significantly promoted the expression of PAR2 in the 16HBE cells (*p* < 0.001), while an EB-A administration at this concentration obviously inhibited the expression of PAR2 (*p* < 0.01). In the co-culture system, the PAR2 activation significantly stimulated the expression of DC surface markers (MHC Class Ⅱ, CD40, CD80, and CD86) (*p* < 0.001, or *p* < 0.01); however, an EB-A intervention significantly inhibited the expression of DC surface markers (*p* < 0.001, *p* < 0.01, or *p* < 0.05) (Figure 7C–G). This suggests that EB-A may prevent DC maturation by inhibiting PAR2 activation.

## 3. Discussion

The Global Burden of Disease study has identified asthma as the most globally prevalent chronic respiratory disease [20]. In the treatment of asthma, traditional Chinese medicine has always had significant effects [21]. For example, *Ephedra sinica* is found in various therapeutic formulations for asthma and allergic diseases; however, the composition of *Ephedra sinica* is complex and diverse, and it is very important and necessary to clarify its material basis for the treatment of asthma, and to develop effective therapeutic drugs for the elucidation of its efficacy and mechanism of action. In the present study, we found and confirmed the anti-asthmatic activity of EB-A, an amide alkaloid compound extracted from *Ephedra equisetina* Bge., and studied its anti-asthmatic mechanism. An OVA-induced allergic asthma mice model was established to study the intervention effect of EB-A on allergic asthma. Dex, which is mainly used in allergic and autoimmune inflammatory diseases, was used as a positive medicine in this study. Our in vivo experiments of allergic asthma mice demonstrated that, consistent with the Dex-treatment group, the EB-A treatment suppressed the typical features of allergic asthma, such as increased serum total IgE levels, lung inflammation, including inflammatory cells infiltration and cytokine production, airway hyperresponsiveness, airway remodeling, and lung injury. Additionally, from pathological sections of the lung and trachea, it is clear that EB-A can significantly inhibit airway remodeling and the proliferation of tracheal smooth muscle in mice; however, the effect of an EB-A treatment on the airway structure and tracheal smooth muscle was not studied in-depth in this study, and further studies are required.

During an asthma attack, the airway epithelium is structurally and functionally abnormal and more susceptible to inhalation stimuli [22]. Further, during sensitization against a specific allergen, several cell types of the innate and adaptive immune system and various ligand–receptor interactions are triggered [23,24,25]. It has been demonstrated that PAR2 activation is involved in the development of allergic airway inflammation [26,27]. As expected, an inhibition of the PAR2 activation was also observed in our study. Afterward, lung injury and inflammation of the asthmatic mice were suppressed after the EB-A treatment. Subsequently, the allergens traversed the damaged epithelial barriers and were internalized by the DCs, which are located in the airways. DCs have a strong ability to recognize and capture antigens in inflammatory environments and are the only professional APCs that can stimulate the activation and proliferation of naïve T cells [28,29,30]. DCs can also promote immune tolerance, partly through the control of regulatory T cells (Tregs), which are crucial to contain autoimmunity and chronic inflammation [31]. There is already considerable experimental evidence that immature DCs (iDCs) themselves can either convert traditional naïve T cells to assume a Treg phenotype and/or promote the function of existing Tregs [32,33,34]. In this study, we simultaneously measured the levels of DCs and Tregs in the peripheral blood and lung tissues of mice, and found that the EB-A treatment inhibited the DC maturation and increased Treg differentiation at the same time.

Mast cells originate from hematopoietic stem cells, circulate in the blood and lymphatic system, migrate to mucosal tissues (such as epithelial cells, glands, smooth muscle cells), and then develop and mature under the action of a tissue-specific microenvironment [35,36]. They are thought to be a key driver of long-term pathophysiological changes and tissue remodeling related to chronic allergic inflammation in asthma [37]. When the body is re-exposed to the same allergen, cross-linking of a high-affinity IgE receptor (FcεRI) on the surface of MCs with multivalent antigens rapidly leads to mast cells activation and the release of a large number of allergic mediators [38]. In this study, we also investigated the effect of EB-A on MCs activation in vivo and in vitro. We showed that EB-A significantly inhibited the mast cell activation and degranulation, including reduced levels in mMCP-1, and a decreased release in tryptase, histamine, and leukotriene C4.

Allergens activate DCs directly through pathogen-associated molecular patterns, but also indirectly by engaging the airway epithelial cells. In our experiments, we observed that the directions of the PAR2 and DCs changes were consistent. To investigate the effect of PAR2 activation in lung epithelial cells on DC maturation, 16HBE cells were co-cultured with BMDCs. The results confirmed that the PAR2 activation stimulated the DCs to mature, as indicated by the increase in the number of surface molecular markers. We also found that DC maturation was inhibited after an EB-A intervention with PAR2. These results suggest that EB-A may prevent the maturation of DCs by inhibiting the PAR2 activation.

## 4. Materials and Methods

### 4.1. Analysis of EB-A

EB-A was previously isolated from the dried grass stem of *Ephedra equisetina* Bge. With 29% methanol as the mobile phase, the EB-A sample was analyzed through the chromatographic column (COSMOSIL, Tokyo, Japan) of the LC-52 semi-preparative liquid analyzer, and the retention time was recorded.

### 4.2. Mouse Model

Female BALB/c mice (*n* = 10 mice per group), purchased from Beijing Vital River Laboratory Animal Technology Co., Ltd. (SCXK (Jing) 2021–0006; Beijing, China), were used for all experiments. The mice were housed in a ventilated cage system. The protocol was approved by the Ethics Committee on Animal Experimentation of Henan University of Chinese Medicine (DWLI2018080003, 15 August 2018–15 August 2023), and the study was carried out under the approved guidelines. Except for the normal control (NC) group, the other mice were used in the preparation of the allergic asthma model. During the sensitization period, the mice were intraperitoneally injected with 0.2 mL of sterile saline containing 50 µg of OVA and 0.05 mL of alum adjuvant on days 0, 7, and 14, respectively. One week after the last sensitization, the mice were challenged by an aerosolized inhalation of 1% OVA for 30 min/day from day 21 to day 23.

### 4.3. Treatment

The mice treated as described above were divided into a model (OVA) group, a positive drug (Y, 0.5 mg/kg/day) group, and EB-A treatment with low (Low, 10 mg/kg/day) and high dose (High, 20 mg/kg/day) groups. From day 21 to day 23, the mice in the treatment groups received EB-A or dexamethasone acetate (Dex) once per day via oral gavage. The mice in the normal control (NC) group were challenged and treated with the same volume of normal saline.

### 4.4. Cough and Wheeze Test

When the last nebulization for the asthma challenge finished 24 h, the cough and wheeze indexes were detected by a multifunctional cough and wheeze–inducing device. For the detection of the cough index, referring to the previously reported method with a slight modification [39], the mice were placed in the device filled with 25% ammonia water for 20 s to induce coughing. The incubation period (i.e., time to first cough after induction) and the number of coughs within 2 min in each mouse were recorded.

Analogously, an equal volume mixture of 2% acetylcholine chloride and 0.1% histamine phosphate was used for inducing wheezing, and the incubation period and number of wheezes within 2 min were recorded. Finally, differences in the number and incubation period of a cough and wheeze of the mice in each group were analyzed.

### 4.5. Airway Hyperresponsiveness (AHR)

When the last nebulization for the asthma challenge finished 24 h, the AHR was measured on the groups of mice using DSI’s unconstrained whole-body plethysmography (WBP) (Data Sciences International, Inc., Saint Paul, MN, USA) in response to increasing concentrations of methacholine (MeCh) (0, 3.125, 6.25, 12.5, 25, and 50 mg/mL). The Penh value was used to reflect the degree of AHR. The FinePointe software was used for the data analysis.

### 4.6. Collection and Measurement of Serum and Broncho-Alveolar Lavage Fluid (BALF)

Immediately following the assessment of AHR, blood samples were collected from all experimental animals by extracting their eyeballs. After centrifugation at 4000 rpm for 10 min, the serum was collected and stored at −80 °C. Subsequently, the BALF was obtained by inserting a tracheal tube and performing a lung lavage (thrice) with 0.5 mL of sterilized normal saline. The collected BALF was centrifuged at 3000 rpm at 4 °C for 10 min. Then, the supernatants were harvested and stored at −80 °C for subsequent cytokine measurements.

Next, the IgE, PGD2, HIS, and LTC4 in the serum, and IL-4, IL-5, IL-10, IL-13, INF-*γ*, TNF-*α*, mMCP-1, and *β*-MCT in the serum and BALF were analyzed by ELISA following the manufacturer’s instructions.

### 4.7. Lung Histopathology

For histological analysis, the lungs and tracheas were fixed in 4% paraformaldehyde for at least 48 h, embedded in paraffin, and cut for hematoxylin and eosin (HE) and Masson staining. The histological score was calculated blindly based on the bronchial morphology and inflammation as previously described [40]. The collagen fiber area in the Masson staining was calculated using Image-Pro Plus 6.0.

### 4.8. Immunofluorescence Staining

After dehydration and a blocking treatment, the lung tissue sections were incubated overnight at 4 °C with primary antibodies (TPSAB1 (1:200, 13343-1-AP, Proteintech, Wuhan, China), PAR2 (1:200, ab180953, Abcam, London, UK), GM-CSF (1:200, 17762-1-AP, Proteintech, Wuhan, China), IL-33 (1:200, 66235-1-Ig, Proteintech, Wuhan, China), and TSLP (1:200, ab47943, Abcam, London, UK)). The next day, the sections were treated with a corresponding fluorescently-labeled secondary antibody (Cy3 conjugated goat anti-rabbit IgG (1:300, GB21303, Servicebio, Wuhan, China) or Cy3 conjugated goat anti-mouse IgG (1:300, GB21301, Servicebio)) for 1 h at 25 °C, and were subsequently mounted using a VECTASHIELD mounting medium containing DAPI. Fluorescence images were acquired using the Nikon Eclipse C1 and Nikon DS-U3 systems (Nikon, Tokyo, Japan).

### 4.9. Western Blot Analysis

A Western blot analysis was performed to detect the expression levels of the TPSAB1 (13343-1-AP, Proteintech, Wuhan, China), PAR2 (ab180953, Abcam, London, UK), GM-CSF (17762-1-AP, Proteintech, Wuhan, China), IL-33 (66235-1-Ig, Proteintech, Wuhan, China), and TSLP (ab47943, Abcam, London, UK). Proteins from the lung tissue were extracted using a mammalian protein extraction kit (Beijing Solarbio, Life Science, Beijing, China) and quantified using a BCA protein assay kit (Solarbio, Life Science, Beijing, China). The protein samples (40 μg) from each group were separated by 10% sodium dodecyl sulfate–polyacrylamide gel electrophoresis (SDS-PAGE) and transferred to a polyvinylidene fluoride (PVDF) membrane. After membrane locking with nonfat milk for 1.5 h at room temperature, the membranes were incubated overnight at 4 °C with primary antibodies, followed by a secondary antibody (goat anti-rabbit 925-68071, goat-mouse 925-32210, Li-COR, Lincoln, NE, USA) for 1 h at room temperature. The density of the proteins was quantified using Odyssey (Clx, Li-COR, Lincoln, NE, USA).

### 4.10. Cell Culture

The RBL-2H3 mast cell line and the 16HBE were purchased from ATCC (Maryland City, MD, USA), and, respectively, cultured in a DMEM or RPMI-1640 medium supplemented with 10% fetal bovine serum (FBS).

BMDCs were isolated and cultured from the female BALB/c mice tibias and femurs. The cells were counted and cultured for 8 days in nontreated Petri dishes with 10 mL of complete growth medium (RPMI, 1% penicillin/streptomycin, 1% NaPy, 1% HEPES, 10% FBS, 100 μL/L *β*-mercapto ethanol, and 1% L-glutamine), supplemented with 20 ng/mL of recombinant murine GM-CSF (Peprotech, Rocky Hill, NJ, USA), and then incubated at 37 °C with 5% CO_2_ with half-volume medium changes every other day. The cells were harvested on day 8 to verify their purity by flow cytometry (FCM) and were used in the subsequent experiments.

### 4.11. Degranulation Assay

We loaded 5 × 10^4^ RBL-2H3 cells in the logarithmic growth phase into each well of a 24-well plate, followed by a 24 h cell culture. Then, they were divided into the normal control group (NC), model group (M, C48/80, 20 μg/mL), different concentrations of EB-A group (e.g., 20 μg/mL C48/80 plus 1 μM, 10 μM, 20 μM, 40 μM, and 80 μM EB-A), and total enzyme group (TE, 1% Triton X-100). After 30 min of incubation in the environment of 37 °C with 5% CO_2_, the supernatant was centrifuged to measure the release of *β*-hexosaminidase (*β*-Hex) and histamine (HIS).

For the assay of the *β*-Hex release, 50 μL of the cell supernatant or lysis buffer were mixed in with 50 μL of a 1 M citrate buffer (pH 4.5) with 1 mM of 4-nitrophenyl N-acetyl-*β*-D-glucosaminide for 1 h at 37 °C, before 150 μL of the stop reagent (1 M Na_2_CO_3_/NaHCO_3_, pH 10.7) was added. The optical density (OD) value of each well at 405 nm was measured by a microplate reader. For the assay of the HIS release, we used a histamine ELISA kit, strictly in accordance with the instructions. We measured the OD value of each well at a wavelength of 450 nm, using a microplate reader (BioTeK, Winooski, VT, USA).

The *β*-Hex release rate (%) = (the OD value of the experimental supernatant—OD value of a blank well)/(OD value of the total enzyme well supernatant—OD value of a blank well) × 100%.

### 4.12. Neutral Red Staining

The cells were treated as indicated in 24-well plates for image capturing. After fixation with 4% paraformaldehyde, 300 μL of a 0.5% neutral red dye was added to the wells for 10 min at room temperature. The cells were washed thrice with 1× PBS and observed by a light microscope.

### 4.13. In-Cell Western Analysis

We loaded 3 × 10^4^ 16HBE cells in the logarithmic growth phase into each well of a 96-well plate, 200 μL/well. After the cells adhered to the wall, they were cultured in a serum-free medium for 24 h. The cells were given different concentrations of PAR2-activating peptide (PAR2-AP) (e.g., 0, 10, 50, 100, 250, and 500 mM). After 24 h of treatment, the expression of PAR2 was detected using an in-cell Western assay to determine the conditions of the PAR2 activation. Then, the cells were seeded into 96-well plates again, according to the above method, and divided into the normal control (NC) group, PAR2-AP treatment group (500 mM), and EB-A intervention group (10 μM). After 24 h of treatment, the expression of PAR2 was detected using an in-cell Western assay.

### 4.14. 16HBE and BMDCs Co-Culture

A co-culture system was constructed using transwell chambers and 24-well culture plates. First, 16HBE cells in the logarithmic growth phase were inoculated into a 24-well culture plate (lower chamber) at 7 × 10^4^/mL, 600 μL/well. After the cells adhered to the wall, they were cultured in a serum-free medium for 24 h. A PAR2-AP treatment for 12 h activated the PAR2 in 16HBE cells, and the AZ3451 or EB-A treatment group was pretreated for 1 h. Subsequently, BMDCs cultured on day 8 were seeded in the transwell chamber (upper chamber) at a concentration of 1 × 10^6^/mL, 100 μL/well. We were careful not to create air bubbles at the interface between the upper and lower chambers. The phenotype of BMDCs in the upper chamber was detected by FCM after 48 h of co-culture.

### 4.15. FCM

The apoptosis and ROS levels in lung tissue homogenized to a single-cell population were assessed using a PE Annexin V apoptosis detection kit Ⅰ (BD Biosciences 7310941, USA), and DCFH-DA (CA1410; Beijing Solarbio Science & Technology Co., Ltd., Beijing, China), respectively, in accordance with the manufacturer’s instructions. An FCM (FACS Aria III, BD Biosciences, New York, NY, USA) was performed to detect a minimum of 10,000 cells.

For the surface staining, single-cell suspensions were incubated with fluorochrome-conjugated antibodies cocktails for 30 min at room temperature in the dark. For the intracellular cytokine staining, the cells were fixed and permeabilized using a Fixation/Permeabilization Buffer Set (eBioscience) in accordance with the manufacturer’s instructions before staining. The fluorochrome-conjugated anti-mouse monoclonal antibodies used in these experiments included the following: CD11c-PE, CD11c-FITC, CD40-PE, CD80-APC, CD86-FITC, MHC Class Ⅱ-FITC, CD4-FITC, CD25-PE, and Foxp3-APC. A fixable viability dye eF780 was used to distinguish the viable cells from nonviable cells. All antibodies were purchased from eBioscience (Thermo Fisher Scientific, Waltham, MA, USA) unless otherwise specified. The flow cytometry data were collected and analyzed using the DIVA software (BD Biosciences, New York, NY, USA).

### 4.16. Molecular Docking

The 2D/3D structure of the EB-A was prepared by the Chemoffice software. The PAR2 protein was used as the receptor (PDB ID: 5NDD) and obtained from the website (https://www.rcsb.org/, accessed on 20 May 2022). The molecular docking was performed using the AutoDock Vina software, and the docking results were further visualized with a 2D/3D molecular descriptor using the Discovery Studio Visualizer software.

### 4.17. Data Analysis and Statistics

The statistical analysis was performed using SPSS 20.0 (IBM, Armonk, NY, USA), and the results were presented as the mean ± standard deviation (SD). A one-way ANOVA was used to determine the differences between every two groups among all, and *p* values less than 0.05 were considered statistically significant. All the statistical analysis results were presented using GraphPad Prism 8.0 (GraphPad Software Inc., San Diego, CA, USA).

## 5. Conclusions

In summary, we identified an amide alkaloid (EB-A) with anti-asthmatic activity from *Ephedra sinica*, and demonstrated the improvement of lung inflammation in asthmatic mice in vivo, as well as the inhibition of DC maturation and MC activation in vivo and in vitro. Moreover, molecular docking and in vitro co-culture experiments indicated that the molecular mechanism of EB-A’s anti-asthmatic activity might be mediated by PAR2. Our study provides powerful experimental data for the elucidation of the active components of *Ephedra sinica*, and contributes to the analysis of the complete pharmacodynamic components of *Ephedra sinica*.

## Data Availability

The data presented in the study are included in the article/Supplementary Materials. Further inquiries can be directed to the corresponding authors.

## Supplementary Materials

The following supporting information can be downloaded at: https://www.mdpi.com/article/10.3390/ijms232113541/s1, Figure S1: the whole uncropped images of the original Western blots with each protein repeated three times; Table S1: the quantitative analysis results of the original Western blots; Table S2: comparison table of target and control bands used for one comparative analysis.
