# Supplementary material for: An Amide Alkaloid Isolated from Ephedra sinica Ameliorates OVA-Induced Allergic Asthma by Inhibiting Mast Cell Activation and Dendritic Cell Maturation
Source: Int J Mol Sci. 2022 Nov 4;23(21):13541. doi: 10.3390/ijms232113541 (PMC9655655; doi:10.3390/ijms232113541)
Supplement: Supplementary file 1 [file ijms-23-13541-s001.zip › ijms-1980779-supplementary.pdf]

An amide alkaloid isolated from *Ephedra sinica* ameliorates OVA-induced allergic asthma by inhibiting mast cell activation and dendritic cell maturation

Supplementary Materials:

Figure S1. The whole uncropped images of the original western blots with each protein repeated three times. Panel A-F represent the whole uncropped images of the original western blots shown in **Figure 4B** and **Figure 6E** with each protein repeated three times, as shown in Test 1, Test 2, Test 3. A, PAR2; B, GM-CSF; C, IL-33; D, TSLP; E, $TPSAB_1$; F, β-actin. NC: nomal control group (referred to as NC in this study); OVA: model group (referred to as OVA in this study); Y: positive control drug (referred to as Y in this study, 0.5 mg/kg/day); Low: low dose group (referred to as Low in this study, 10 mg/kg/day); High: high dose group (referred to as High in this study, 20 mg/kg/day).

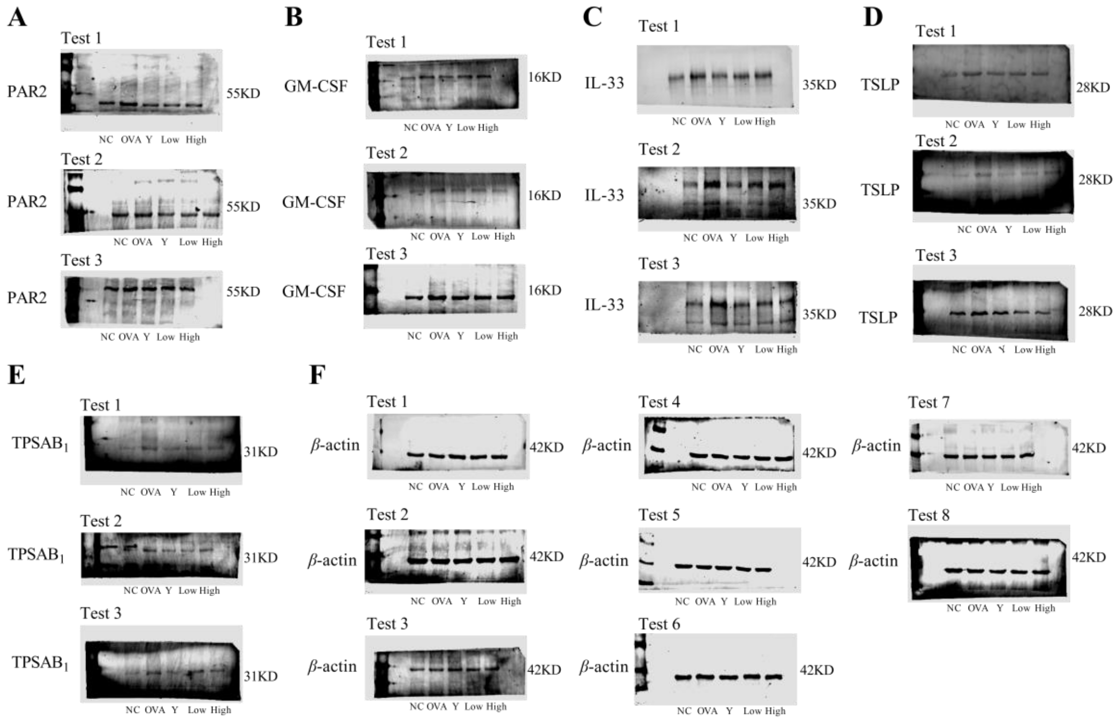

Table S1. The quantitative analysis results of the original western blots. Panel A, B, C, D, E, and F represent the quantitative analysis results of the original western blots shown in **Figure S1** and **Figure 4B & Figure 6E**, from which figures have been derived. And include the results of statistical analysis of the relative expression of each

protein, as shown in item Mean±SD in the tables. A, PAR2; B, GM-CSF; C, IL-33; D, TSLP; E, $TPSAB_1$; F, β-actin. Data are mean ± SD. $n = 3$ per group. $^{\#}p < 0.05$, $^{\#\#}p < 0.01$, $^{\#\#\#}p < 0.001$, compared with the NC group; $^{*}p < 0.05$ vs OVA group. $^{**}p < 0.01$ vs OVA group. $^{***}p < 0.001$, compared with the OVA group. NC: nomal control group (referred to as NC in this study); OVA: model group (referred to as OVA in this study); Y: positive control drug (referred to as Y in this study, 0.5 mg/kg/day); Low: low dose group (referred to as Low in this study, 10 mg/kg/day); High: high dose group (referred to as High in this study, 20 mg/kg/day); DPI : dots per inch.

A

Groups	PAR2 (DPI)			PAR2/*β*-actin
	Test 1	Test 2	Test 3	Mean±SD
NC	87.7	74.9	576	1.00±0.00
OVA	139	115	795	$1.68\pm0.13^{\#\#}$
Y	69.9	69.2	681	$1.12\pm0.30^{**}$
Low	74.3	88.1	632	$1.20\pm0.30^{*}$
High	94	99.2	694	$1.22\pm0.15^{*}$

B

Groups	GM-CSF (DPI)			GM-CSF /*β*-actin
	Test 1	Test 2	Test 3	Mean±SD
NC	81.5	27.6	172	1.00±0.00
OVA	128	46	271	$1.70\pm0.03^{\#\#\#}$
Y	83.2	25.6	207	$1.13\pm0.34^{***}$
Low	114	27.2	196	$1.24\pm0.10^{**}$
High	113	33.1	201	$1.23\pm0.10^{**}$

C

Groups	IL-33 (DPI)			IL-33/*β*-actin
	Test 1	Test 2	Test 3	Mean±SD
NC	324	59.2	59.9	1.00±0.00
OVA	587	160	164	$2.50\pm0.56^{\#\#\#}$

Y	300	54.3	68.4	1.12±0.29***
Low	409	65.8	82.8	1.44±0.28**
High	453	72.4	91.2	1.48±0.22**

D

Groups	TSLP (DPI)			TSLP /*β*-actin
	Test 1	Test 2	Test 3	Mean±SD
NC	133	33.3	105	1.00±0.00
OVA	304	62.9	189	2.27±0.42##
Y	171	17.0	134	1.37±0.77*
Low	159	25.6	89.9	1.20±0.36*
High	152	50.1	60.1	1.24±0.32*

E

Groups	$TPSAB_1$ (DPI)			$TPSAB_1$/*β*-actin
	Test 1	Test 2	Test 3	Mean±SD
NC	16.2	9.6	9.7	1.00±0.00
OVA	67.1	41.0	41.2	3.93±0.48###
Y	35.5	23.8	20.1	2.11±0.51***
Low	49.2	24.3	22.9	2.55±0.64***
High	42.4	31.5	26.3	2.77±0.04**

F

Groups	*β*-actin (DPI)							
	Test 1	Test 2	Test 3	Test 4	Test 5	Test 6	Test 7	Test 8
NC	780	639	139	1400	4260	520	886	333
OVA	979	594	137	1180	3740	526	856	270
Y	1240	505	112	1120	3920	517	834	213
Low	1030	513	130	1110	3230	494	775	221
High	924	565	134	1330	3430	497	853	202

Table S2. Comparison table of target and control bands used for one comparative abalysis.

Class	*β*-actin

		Test1	Test2	Test3	Test4	Test5	Test6	Test7	Test8
PAR2	Test1						√		
	Test2				√				
	Test3				√				
GM-CSF	Test1					√			
	Test2							√	
	Test3		√						
IL-33	Test1							√	
	Test2						√		
	Test3		√						
TSLP	Test1				√				
	Test2						√		
	Test3								√
$TPSAB_1$	Test1						√		
	Test2	√							
	Test3			√					

[√]The two corresponding bands are the target and control band used for one comparative abalysis.
